# The putative role of the microbiota in the development of neuropsychiatric disorders following early childhood malnutrition

**DOI:** 10.1080/19490976.2026.2643373

**Published:** 2026-03-17

**Authors:** Yahya Jama, Waliul Khan, Stephen M. Collins

**Affiliations:** a Department of Medicine, The Farncombe Family Digestive Health Research Institute, Faculty of Health Sciences, McMaster University, Hamilton, Ontario, Canada

**Keywords:** Gut–brain axis, brain development, cognitive function, psychiatric disorders, depression, attention deficits, microbiota-directed foods, short chain fatty acids, phospholipids

## Abstract

Early childhood malnutrition (ECM) is robustly associated with increased risk of cognitive impairment and neuropsychiatric disorders across the lifespan, yet the biological mechanisms underlying this vulnerability remain incompletely defined. Accumulating clinical evidence indicates that ECM is associated with delayed maturation and reduced diversity of the intestinal microbiota, including depletion of taxa involved in short-chain fatty acid production and complex carbohydrate fermentation. These microbial alterations coincide with broader metabolic, immune, and barrier dysfunctions – such as reduced availability of neuroactive metabolites, low-grade inflammation, and impaired intestinal and vascular integrity – that plausibly intersect with critical processes in brain development. Experimental studies in animal models demonstrate that perturbation of microbiota-derived signaling during sensitive early periods is sufficient to induce lasting neurodevelopmental and behavioral changes, providing proof of concept for a causal role. However, in human populations, the microbiota remains best viewed as a biologically plausible intermediary rather than a proven determinant of outcome. Future progress will require integrative longitudinal studies and developmentally timed interventions to test whether restoration of microbiota function can modify neurodevelopmental trajectories. Clarifying these relationships has important implications for understanding the long-term consequences of early nutritional adversity and for identifying preventive strategies in settings where ECM remains prevalent.

## Introduction

The Barker hypothesis holds that early-life insults can induce lasting physiological and metabolic changes, establishing the field of developmental origins of health and disease (DOHaD).^1^ Among these insults, inadequate nutrition has emerged as a critical and potentially modifiable determinant of child development.

Malnutrition is broadly defined as an imbalance between the supply of protein and energy and the body's demand for them to ensure optimal growth and function.^2^ Childhood undernutrition encompasses both macro- and micro-nutrient deficiencies and is often referred to as acute malnutrition (AM).^3^ Protein–energy deficiency may be primary or secondary to an underlying disease. Primary acute malnutrition is prevalent in low- and middle-income countries and closely associated with poor socioeconomic and environmental conditions.

Early childhood malnutrition (ECM) refers to children under the age of 5 and initially presents as stunting (low height-for-age) or wasting (low weight-for-height) and can progress to marasmus or kwashiorkor.^4^ United Nations Children's Fund (UNICEF)^5^ Marasmus, the more common form, represents an adaptive response to starvation, characterized by profound muscle wasting and fat loss due to global nutrient deprivation. By contrast, kwashiorkor reflects a maladaptive response to insufficient protein intake despite adequate caloric consumption and is distinguished clinically by the presence of edema. ECM constitutes a major global health crisis, accounting for an estimated 45% of deaths among children under five. (United Nations Children's Fund (UNICEF)^5^ Evidence from famine cohorts and large-scale longitudinal studies demonstrates a strong association between early-life undernutrition and later development of neuropsychiatric disorders.^6‐8^ A causal relationship is suspected, given the critical dependence of brain development on adequate nutrition. Involvement of the microbiota is postulated via the microbiota‒gut‒brain axis. We acknowledge the importance of prenatal factors, including maternal nutrition, on brain development,^9^ but this review focuses on the postnatal microbiota in the ECM, its impact on the brain, and the clinical evidence linking the ECM to the development of cognitive and mood disorders across the lifespan.

## Literature review methodology

A literature search was conducted in PubMed for articles published between January 1950 and February 2025. The search strategy combined the following keywords: malnutrition, undernutrition, protein deprivation, famine, infant, early life, development, cognition, depression outcomes, gut microbiome/microbiota, gut-microbiota-brain axis, probiotics, nutritional supplementation, and animal models. We included prospective cohorts or retrospective famine studies that met specific criteria: a sample size of at least 50, follow-up from infancy to adulthood or retrospective assessment in adulthood, and reporting on cognitive, emotional/behavioral, or growth outcomes.

## Results

### Time courses of human brain and microbiota development in humans

The first 3 y of life constitute a critical, highly plastic period of brain development, marked by rapid neuronal growth, synaptogenesis, synaptic pruning, and myelination. By age three, brain volume reaches approximately 80% to 90% of its adult size (Table 1). During infancy, sensory, limbic, and associative cortical circuits develop most rapidly, followed by refinement of executive networks in the toddler period.^10^^,^^11^ These neurodevelopmental processes are strongly influenced by experience and environmental factors and coincide with the establishment and maturation of the gut microbiota. Concurrently, the infant gut microbiota transitions from a low-diversity, milk-adapted community dominated by Bifidobacterium and facultative anaerobes in early infancy to a more diverse, adult-like composition enriched in Bacteroides and Firmicutes after weaning.^12‐14^ This maturation influences immune system development, metabolic signaling, including the production of short-chain fatty acids (SCFA), and the availability of neuroactive metabolites. These effects occur during a period when microglial function, blood–brain barrier integrity (BBB), and synaptic pruning are particularly responsive to peripheral signals, including those from the microbiota.^15^^,^^16^ Disruption of microbiota development during this window (Table 2) due to factors such as malnutrition, infection, or antibiotic exposure has been identified as a biological contributor to long-term neurodevelopmental and behavioral vulnerability.

**Table 1.** Early postnatal brain development and reversibility.

Age (months)	Neurodevelopment	Reversibility
0–3	Explosive synaptogenesis in primary sensory cortices, initial sensory map calibration; Brainstem and subcortical myelination	Mostly irreversible
3–6	Peak synaptogenesis in visual cortex; Sensory integration; Rapid cerebellar growth	Mostly irreversible
6–9	Acoustic discrimination; Strengthening of long-range connections; Increased limbic engagement	Partially reversible
9–12	Accelerated hippocampal maturation; Consolidation of attachment circuits; Emergence of stress-regulation set-points e	Partially reversible
12–18	Peak synaptic density in many cortices Onset of synaptic pruning. Rapid frontal myelination	Structurally irreversible Functionally modifiable
18–24	Peak synaptic density in many cortices; Onset of synaptic pruning; Rapid frontal myelination	Structurally irreversible Functionally modifiable
24–36	Accelerated pruning and specialization; Improved PFC–limbic integration; Executive function emergence	Functionally highly plastic

From birth to 36 months, brain maturation proceeds through waves of synaptogenesis, myelination, pruning, and circuit specialization. The first 6 months are dominated by explosive sensory cortical synaptogenesis and early subcortical myelination, processes that are largely structurally irreversible. Between 6 and 24 months, long-range connectivity, hippocampal maturation, and frontal development advance amid active pruning, permitting functional modification despite structural constraint.

**Table 2.** Development of human microbiota.

Age	Dominant Taxa	Functional implications
0–7 d	*Lactobacillus* (*L. crispatus*, *L. gasseri*), *Bifidobacterium*, *Bacteroides*, *Prevotella*,	Rapid anaerobic conditioning; early acidification
1–4 weeks	*Bifidobacterium longum* subsp. *infantis*, *B. breve*, *B. bifidum*; low *Enterobacteriaceae*	HMO metabolism; acetate/lactate. High acidity
1–4 months	Dominant *Bifidobacterium*; minor *Escherichia coli*, *Streptococcus*	Treg induction; reduced intestinal permeability
3–6 months	*Veillonella*, *Clostridium sensu stricto*, *Eubacterium*, early *Ruminococcus*	Expansion of carbohydrate fermentation; early SCFA diversity
6–9 months	*Bacteroides fragilis*, *B. thetaiotaomicron*, *Akkermansia muciniphila* (low abundance).	Mucus utilization; immune–epithelial cross-talk
9–12 months	*Faecalibacterium prausnitzii*, *Roseburia*, *Lachnospiraceae*	Butyrate production; anti-inflammatory tone
12–24 months	Stable Firmicutes–Bacteroidetes balance; ↑ diversity	Adult-like SCFA profile; bile acid transformation
12–36 months	*Faecalibacterium*, *Ruminococcaceae*, *Bacteroides*	Mature immune tolerance; gut–brain signaling [10‐16]

From birth to 36 months, the intestinal microbiota undergoes staged ecological succession. Early colonization is dominated by Lactobacillus and Bifidobacterium species that metabolize human milk oligosaccharides and promote anaerobic conditioning. With complementary feeding, fermentative taxa expand, increasing short-chain fatty acid production and immune–epithelial cross-talk. By 12–36 months, greater diversity and stabilization of community structure support bile acid metabolism, immune tolerance, and gut–brain signaling.

Time course of development of the human microbiota at the taxi level during the first 3 y of life. These data are applicable to infants following vaginal delivery.

### Compositional changes in the microbiota in humans experiencing early childhood malnutrition

Geography, diet, infection burden, antibiotic exposure, age, and breastfeeding are major determinants of early-life gut microbial ecology. Settings with high enteropathogen exposure show recurrent Proteobacteria blooms, whereas breastfeeding sustains Bifidobacterium dominance in infancy, even in some undernourished cohorts.^17‐19^ Clinical phenotypes – stunting, wasting, and kwashiorkor – share overlapping yet distinct microbial signatures. In the Malawi twin study, microbiota from the malnourished twin alone transmitted weight loss and altered both bacteriome and virome in recipient mice, supporting a causal contribution.^20^

Across cohorts, moderate and severe malnutrition are consistently associated with reduced microbial diversity relative to healthy peers, and lower diversity correlates with impaired growth indices.^17^^,^^20^^,^^21^ Community compositional shifts often exceed age-related variation within the same environment.^20^^,^^21^ Butyrate- and fiber-fermenting taxa – including Faecalibacterium, Roseburia, Eubacterium, and Ruminococcus are frequently depleted, accompanied by reduced fecal butyrate and impaired carbohydrate fermentation.^18^^,^^21^^,^^22^ Concurrently, Proteobacteria (e.g., Escherichia/Shigella) expand, particularly during diarrheal or infectious episodes, contributing to ecological instability.^20^^,^^21^ Undernourished children also show increased stool abundance of oral/upper-gastrointestinal (GI) taxa (Streptococcus, Veillonella) and mucin-degrading organisms such as Akkermansia, consistent with a shift toward host-derived substrates under dietary constraint.^19^^,^^23^ Metabolically, lower fecal butyrate and propionate are among the most reproducible signatures of malnutrition, aligning with loss of SCFA-producing taxa.^22,24‐27^ Plasma and fecal metabolomics further demonstrate perturbed amino acid profiles (including reduced essential amino acids) and altered bile acid pools, reflecting impaired host nutrient status and microbial metabolic capacity.^20^^,^^22^ Importantly, integrated taxonomic–metabolomic metrics, such as microbiota maturation indices and SCFA levels, correlate more strongly with anthropometry and clinical recovery than taxonomy alone, underscoring the functional dimension of dysbiosis in malnutrition.^17^^,^^18^^,^^22^

Limitations of these studies include methodological differences and cohort heterogeneity. Species/strain reporting varies by method (16S rRNA vs. shotgun metagenomics vs. culture): older 16S studies report genus-level changes, whereas newer shotgun metagenomic studies and culture-based work provide strain-level and functional resolution. Interpret findings with attention to methodology.^17^^,^^21^^,^^23^ Cohort heterogeneity limits any single universal taxonomic signature – the reproducible motifs are immaturity, lower diversity, and functional SCFA loss rather than identical lists of increased/decreased species across all settings.^18^^,^^19^

### Review of human studies linking early childhood malnutrition to later neuropsychiatric disorders

Early-life undernutrition is associated with the development of behavioral abnormalities later in life (Table 3). The Barbados Nutrition Study (BNS) is a 57-y longitudinal investigation of children exposed to moderate-to-severe protein–energy malnutrition (marasmus and kwashiorkor) in infancy.^28^ Two cohorts of Barbadian children, matched for age, sex, and socioeconomic background, were followed. By age 5, previously malnourished children displayed elevated depressive symptoms.^28^^,^^29^ Although some social symptoms improved, depressive symptoms persisted.^29^^,^^30^ In later life, individuals with early malnutrition showed higher neuroticism and lower levels of extraversion, openness, and conscientiousness.^31^^,^^32^ The Jamaica Study (JS) is a 30-y randomized controlled trial that followed stunted children and healthy peers.^33^ Stunted children were randomly assigned to receive nutritional supplementation, psychosocial stimulation, both, or neither. In toddlerhood, stunted children exhibited reduced happiness and increased fussiness, which later predicted neurobehavioral delays.^34^ By ages 17–18, depressive symptoms and lower self-esteem were reported more frequently among stunted adolescents^34^^,^^35^ with neurobehavioral changes persisting into adulthood.^7^ Analyzes of the China Health and Retirement Longitudinal Study (CHARLS) revealed a dose-dependent association between famine exposure and depression risk.^36‐38^

**Table 3.** Long-term outcomes after early-life undernutrition.

Study cohort	Age of neuropsychiatric dysfunction	Dysfunction
Barbados Nutritional Study (8)	Childhood; Adolescence (Adulthood (Early-Middle)	Lower cognition (IQ) and Depression symptoms across follow-ups [28‐32] Attention deficits persisting into Adulthood [28,30,39,40] Lower educational achievements into adulthood [29,30,39‐41] Altered personality traits in adulthood [32,42] Reduced socioeconomic outcomes in adulthood [43]
The Jamaica Early Childhood Stimulation Trial	Childhood; Adolescence Adulthood (Early - Middle)	Nutritional Supplementation had no sustained cognitive or psychiatric benefits [33‐35] Psychosocial stimulation produced significant lasting gains in IQ, attention, and psychosocial functioning into adulthood, still below non-stunted group [7,35] non-stimulated stunted children showed persistent deficits: lower cognition, higher depression, sustained attention problems, and poorer educational achievement into adulthood [7,35,44,45]
The China Health and Retirement Longitudinal Study	Adulthood (Middle-Late)	In utero famine exposure increased risk for depression and cognitive deficits in late adulthood a dose-dependent manner [36,38] Early to middle childhood exposure increased risk for depression and cognitive deficits in late adulthood [36‐38] Exposure in-utero and in early childhood, increased risk for attention related deficits in late adulthood

Data from the Barbados Nutrition Study, the Jamaica Early Childhood Stimulation Trial, and the China Health and Retirement Longitudinal Study show persistent neurocognitive and psychiatric sequelae of early malnutrition or prenatal famine exposure. Reported outcomes include lower cognition, attention deficits, depressive symptoms, reduced educational attainment, and adverse socioeconomic status into adulthood. Nutritional supplementation alone showed limited sustained benefit, whereas early psychosocial stimulation improved, but did not normalize, long-term outcomes.

The most severe effects were observed among those exposed in utero. Individuals exposed to famine between the ages of 6 and 13 were at heightened risk of developing depression later in life.^37^^,^^38^ Comparable patterns were observed across cohorts in the Philippines, South Africa, Mauritius, Guatemala, Peru, and Brazil.^46‐50^ Studies on the in-utero survivors of the Dutch famine during WWII showed a consistent link to an increased risk of schizophrenia, antisocial personality disorder, and major affective disorders later in adult life.^51‐55^

Cognitive deficits were identified in children and adults following early-life undernutrition. In the BNS, children malnourished in infancy showed lasting impairments in memory, attention, social functioning, and academic achievement.^28‐30,41,56^ The JS cohort similarly demonstrated stunting-related delays by age two that persisted through late childhood.^33^^,^^34^ In the BNS, attention deficits in childhood were independently associated with infant malnutrition and predicted poor adolescent school performance.^41^^,^^56^ The JS cohort similarly showed lower digit-span scores, a measure of cognitive function, among stunted children at ages 7–8 and 11–12.^34^^,^^35^^,^^44^ In adulthood, BNS participants had an ADHD diagnosis eight times more often than controls, who had none.^39^ While JS participants showed reduced cognitive function (IQ) compared to controls, this deficit persisted even in those who received nutritional rescue.^7^ Taken together, the results of these clinical studies establish a firm association between early childhood malnutrition and the risk of developing psychiatric disorders, impaired cognitive function, and attention deficit across the lifespan. They provide the basis for exploring underlying mechanisms and the potential role of the microbiota.

### Brain and microbiota development in the mouse

Murine brain development proceeds through tightly timed embryonic and early postnatal phases characterized by neurogenesis, circuit assembly, synaptogenesis, pruning, and myelination. Germ-free, antibiotic-perturbed, and gnotobiotic mouse models demonstrate that early-life microbial signals are required for normal microglial maturation, synaptic refinement, stress-axis programming, and behavioral development, with the greatest sensitivity occurring before weaning. Reconstitution with specific commensal taxa during these windows can rescue behavioral and neurodevelopmental phenotypes, whereas later interventions yield incomplete recovery, underscoring the existence of microbiota-dependent sensitive periods in brain development.^12^^,^^57^^,^^58^

In parallel, murine gut microbiota development follows a stereotyped succession. At birth (P0), the gut is rapidly colonized by facultative anaerobes, including Escherichia and Enterococcus, which consume residual oxygen and enable subsequent anaerobic expansion.^59^^,^^60^ During the early neonatal period (P1–P7), pioneer taxa such as Lactobacillus and Bifidobacterium emerge, microbial diversity remains low, and immune tolerance pathways are initiated.^59‐61^ Between P7 and P14, obligate anaerobes, including Bacteroides and early Clostridia, expand, accompanied by increased microbial metabolic output and host immune crosstalk.^60^^,^^61^ The preweaning interval (P14–P21) is a critical window in which microbial antigen exposure and early short-chain fatty acid production are required for durable immune and neurodevelopmental programming.^15^^,^^57^^,^^61^ Weaning (P21–P28) induces a major ecological shift driven by dietary changes, resulting in Firmicutes expansion, robust SCFA production, and the acquisition of adult-like metabolic capacity. Thereafter, the microbiota becomes increasingly stable and resilient, with early-life perturbations exerting lasting programming effects.^59^^,^^61^^,^^62^

### Alignment of murine and human studies

Findings from murine models showing that early-life malnutrition perturbs microbiota-dependent neurodevelopmental processes align closely with observations from long-term human cohort studies. Natural experiments, such as the Dutch Hunger Winter, further support the existence of sensitive developmental windows, linking prenatal nutritional deprivation to increased risk of neuropsychiatric disorders, including schizophrenia and affective illness.^63^^,^^64^ Analyzes of human birth cohort studies show parallels with emerging murine data, indicating that early-life undernutrition and growth stunting are accompanied by delayed or immature gut microbiota development, reduced abundance of short-chain fatty acid–producing taxa, and disruption of microbiota-mediated immune function.^65‐67^ Together, these research approaches support the microbiota as a plausible mechanistic intermediary linking early-life malnutrition to long-term neurodevelopmental vulnerability.^62^^,^^66^^,^^68^

### Microbial metabolites and the brain

Early-life malnutrition exerts effects on brain development, in part by disrupting the acquisition and function of durable developmentally instructive gut microbes and their metabolites. Convergent murine evidence shows that SCFAs, particularly butyrate, are central mediators linking diet, microbiota, and neurodevelopment. Butyrate promotes neurogenesis, supports synaptic plasticity, maintains blood–brain barrier (BBB) integrity, and suppresses neuroinflammation via epigenetic mechanisms, including histone deacetylase inhibition and G protein–coupled receptor signaling (GPR41/43/109A).^63^^,^^66^^,^^69^

In gnotobiotic and dietary-intervention models, fiber insufficiency during weaning leads to loss of butyrate-producing taxa (Faecalibacterium, Roseburia, Eubacterium), with consequent impairments in synaptic plasticity and BBB function that are moderately durable yet partially reversible with timely dietary rescue.^63^^,^^70^^,^^71^ Superimposed antibiotic exposure further depletes butyrate-producing Clostridium spp., reducing BDNF expression and impairing learning and memory through epigenetic mechanisms.^22^^,^^23^^,^^72^

Parallel pathways involve tryptophan metabolism and serotonergic signaling, which are shaped by microbial catabolism into indoles and kynurenine pathway intermediates by taxa including Bacteroides, Clostridium, and Lactobacillus. Germ-free and antibiotic-perturbed mice exhibit altered central serotonin turnover and hippocampal serotonergic signaling, with some changes resistant to later recolonization, underscoring sensitive developmental windows.^62^^,^^68^^,^^73^ Malnutrition-associated dysbiosis, therefore, plausibly alters the balance between dietary tryptophan availability and microbiota-derived neuroactive metabolites during early neurodevelopment.

Lipid pathways provide an additional mechanistic link. Undernutrition is associated with dyslipidemia and reduced IGF- 1, while depletion of Bacteroides reduces microbial sphingolipid production, lipids required for normal myelination and neuronal membrane integrity.^74‐76^ In murine models, these alterations indirectly impair synaptogenesis and myelination, consistent with an inflammatory bias and reduced trophic support.^75^^,^^76^ Psychosocial stress interacting with malnutrition further disrupts microbe–vagus–oxytocin signaling, particularly through loss of Lactobacillus reuteri, leading to impaired prefrontal synaptic plasticity and social behavior; early microbial reconstitution yields partial rescue.^77^^,^^78^

### Alignment with human data

Although causality for specific taxa cannot be assigned in humans, birth cohort studies consistently show that undernourished children exhibit microbiota immaturity, depletion of SCFA-producing taxa, and altered microbial metabolic capacity.^17^^,^^23^^,^^79^ Together, these findings support a coherent translational model in which early malnutrition disrupts microbiota-derived metabolic, immune, and neuroendocrine signaling – particularly butyrate, tryptophan metabolites, and lipids – during sensitive periods, producing durable neurodevelopmental vulnerability (Table 4).

**Table 4.** Gut microbiota patterns linked to neurodevelopment in childhood undernutrition.

Study	Age	Health status	Associated taxa	Functional implications	Neurodevelopment outcomes
Bangladesh (17)	≤24 months	Moderate-Severe Malnutrition	Altered age-discriminatory taxa (e.g., F. prausnitzii, Ruminococcus spp., Dorea spp., L. mucosae, S. thermophilus, B. longum)	Microbiota immaturity (lower microbiota-for-age) and reduced bacterial diversity in malnourished children	None measured
Bangladesh (128)	12 months (cross-sectional)	Moderate Acute Malnutrition (vs Well-Nourished Controls)	↓ B. fragilis; ↑ S. salivarius; ↑ R. mucilaginosa	Reduced alpha diversity, odd-chain fatty acids and ceramides; lipid/fermentation signatures correlated with Bacteroides (incl. B. fragilis) and with EEG/language measures	In malnourished children, reduced beta/gamma EEG power and lower Bayley expressive communication, and motor domains and reduced Wolke vocalization
Bangladesh (151)	6–36 months (longitudinal/cross-sectional)	Severe Acute Malnutrition (SAM)	↑ B. longum; ↑ S. gallolyticus; ↑ E. coli	Increased microbiota immaturity. The Bifidobacterium-enriched SAM microbiota shows relatively reduced amino-acid and micronutrient biosynthesis and carbohydrate-utilization pathways.	None measured
Indonesia (24)	3–5 y (cross-sectional)	Stunting (vs Non-Stunted)	↓ Prevotella, ↑ Alloprevotella, ↑ Enterobacter; ↓ Bacteroidetes, ↑ Firmicutes	higher *α*-diversity in the stunted children. Prevotella 9 consistent with lower dietary fiber intake. Stunted children had higher fecal SCFA/branched-chain fatty acids concentrations	None measured
Madagascar (129)	2–5 y (cross-sectional)	Moderate–Severe Stunting (vs Non-Stunted)	↑Streptococcaceae	Neurodevelopmental scores associated primarily with stunting and socioeconomic status. ↑Streptococcaceae linked to lower Personal-Social score	Modest link between microbiome *α*-diversity and neurodevelopment, no direct pathway linking stunting, microbiota and neurodevelopment
Central African Republic and Madagascar (152)	2–5 y (cross-sectional)	Moderate–Severe Stunting with Enteropathy-Like Features (vs Non-Stunted)	↑ Streptococcus spp. (S. oralis, S. salivarius, S. mitis, S. pneumoniae), ↑ Veillonella, ↑ Lactobacillus salivarius, ↑ Fusobacterium periodonticum; ↓ Clostridia	Enrichment of upper oral–associated taxa consistent with gastrointestinal barrier dysfunction and small intestinal bacterial overgrowth, potentially contributing to mucosal inflammation. Reduction in butyrate producers, predicted butyrate production trends lower	None measured

Across cohorts from Bangladesh, Indonesia, Madagascar, and the Central African Republic, early-life undernutrition and stunting are consistently associated with microbiota immaturity/dysbiosis, including shifts in age-discriminatory taxa, loss of beneficial taxa (e.g., SCFA producers) and enrichment of potentially pro-inflammatory or oral-derived taxa. Functional implications include reduced microbial diversity, altered lipid/fermentation metabolites, and reductions in butyrate-producing capacity, particularly in enteropathy-like states, suggesting mucosal dysfunction. Direct neurodevelopmental outcomes were measured in only two studies, in which altered microbiota/metabolite profiles were associated with EEG and language measures, whereas most cohorts report no neurodevelopmental testing.

### Microbiota metabolite sensing and access of metabolites to the brain

Sensing of microbial and dietary metabolites in the gut is a key driver of immune, metabolic, and neurodevelopmental processes and is markedly impaired in ECM.^80‐82^ Fermentation of dietary substrates by the gut microbiota generates short-chain fatty acids (SCFAs), which act as central signaling molecules sensed by enteroendocrine cells (EECs) via G-protein–coupled receptors, including GPR41 and GPR43. Activation of these pathways regulates secretion of hormones such as glucagon-like peptide-1 (GLP-1) and peptide YY, which reinforce epithelial barrier integrity, modulate mucin production, and coordinate immune–metabolic signaling.^83^ In parallel, the aryl hydrocarbon receptor (Ahr) serves as a critical mucosal sensor of microbial and dietary tryptophan metabolites, including indoles. Deficiency of Ahr ligands during undernutrition promotes macrophage-driven inflammation with elevated TNF-*α* and IL-6, while suppressing IL-22–mediated epithelial repair by innate lymphoid cells, thereby compromising barrier function.^80^^,^^83^

In ECM, these nutrient-sensing systems are simultaneously blunted by reduced microbial diversity and loss of key fermentative taxa, leading to diminished SCFA availability and impaired downstream immune–metabolic signaling.^81^ The resulting inflammatory milieu, characterized by elevated IL-6 and TNF-*α*, induces hepatic growth hormone resistance, reduces insulin-like IGF-1 synthesis, and suppresses activation of growth-critical pathways such as mTORC1.^84^ These alterations integrate microbial dysbiosis, chronic inflammation, and impaired nutrient sensing into a unified mechanism underlying growth failure. Concurrently, childhood malnutrition is associated with structural and functional disruption of the intestinal barrier, including villus blunting, mucosal inflammation, and reduced absorptive surface area, which co-occur with microbial dysbiosis and increased epithelial permeability.^67^^,^^85^ Once luminal antigens and microbial metabolites cross the compromised epithelium, altered vascular permeability can facilitate systemic dissemination; in kwashiorkor, changes in microvascular function have been reported, and experimental studies demonstrate dysbiosis-induced disruption of the gut vascular barrier via tissue factor– and protease-activated receptor-1–mediated pathways, amplifying systemic inflammation.^86‐89^

Microbial signals also reach the brain through neural and metabolic relays. The enteric nervous system and the vagus nerve transmit microbiota-derived information, with SCFAs modulating vagal afferent activity and influencing affective and metabolic phenotypes; both depressive-like behavior induced by microbiota transfer and the anxiolytic effects of Bifidobacterium longum are partially vagus-dependent in animal models.^90^^,^^91^ Undernourished children exhibit diminished parasympathetic tone and impaired vagal signaling, consistent with reduced gut–brain communication.^92^^,^^93^ Metabolically, undernutrition forces adaptive shifts toward glycogenolysis, muscle proteolysis, lipid utilization, and ketone body production to preserve cerebral energy supply, but at the cost of growth and anabolic processes. Germ-free mouse studies demonstrating transmission of growth retardation following colonization with microbiota from malnourished donors further support a contributory, though not exclusive, role for the microbiota in mediating these systemic and neurodevelopmental adaptations.^23^ Finally, ECM is characterized by combined immunodeficiency and chronic low-grade inflammation, with impaired innate and adaptive immune responses coexisting with elevated CRP and pro-inflammatory cytokines. This paradox likely reflects increased microbial translocation and persistent immune activation^82^^,^^94^^,^^95^

The BBB permits selective passage of small microbiota-derived metabolites, notably SCFAs such as acetate, which cross via monocarboxylate transporters and are required for normal microglial maturation and BBB integrity; germ-free mice exhibit increased BBB permeability that is normalized by microbial colonization or SCFA supplementation.^15^^,^^96^ Other microbial metabolites, including tryptophan-derived indoles and kynurenine pathway intermediates, access the brain and modulate neurotransmission and neuroimmune tone,^68^^,^^97^ whereas larger microbial products such as lipopolysaccharide rarely cross the BBB intact and instead signal indirectly through endothelial activation and cytokine induction.^98^ In parallel, outer membrane vesicles (OMVs) released by Gram-negative bacteria provide a stable, cell-free means of transporting complex microbial signals systemically, however, direct BBB translocation appears limited.^99‐102^ OMVs readily traverse the intestinal epithelium, interact with immune and vascular cells, and modulate endothelial signaling and barrier properties, thereby indirectly shaping central nervous system function. The choroid plexus, forming the blood–cerebrospinal fluid barrier, acts as a critical immune–epithelial interface that responds to circulating microbial metabolites, OMV-induced cytokines, and inflammatory signals by altering leukocyte trafficking and cerebrospinal fluid cytokine composition, with particular relevance during development.^103^^,^^104^ Circumventricular organs lacking a classical BBB provide additional sensing sites for circulating microbial and inflammatory mediators,^105^ while neural and endocrine relays, especially via the vagus nerve and enteroendocrine signaling, transmit microbiota-derived information to the brain without molecular translocation across barriers.^16^^,^^106^ In early-life malnutrition, disruption of microbial composition and metabolite availability is accompanied by impaired intestinal and vascular barrier function, facilitating systemic dissemination of microbial mediators and inflammatory signals that can influence BBB maturation, OMV-mediated immune signaling, and choroid plexus function during sensitive developmental windows.^67^^,^^85^^,^^86^^,^^88^^,^^107^

Collectively, these pathways indicate that microbial effects on brain development are primarily informational and regulatory, mediated through selective permeability, epithelial and immune interfaces (including OMVs), and neural circuits rather than frank barrier breakdown, providing a biologically plausible link between nutritional adversity and long-term neurodevelopmental vulnerability.

### Malnutrition alters brain functions

(a) Microglia

Microglia control synaptic pruning, neural activity, and myelination and their development is influenced by the microbiota.^15^^,^^108^ The brains of undernourished infants exhibit reduced synaptic arborization, shorter and abnormally oriented dendrites, and persistent alterations in EEG activity, with abnormalities extending into adulthood.^109^^,^^110^ Similarly, rodents undernourished early in life show impaired synaptic pruning,^111^ highlighting their role in these long-lasting neural deficits. In addition, the gut microbiota regulates microglial development; in a model of traumatic brain injury, SCFAs, including propionate, acetate, and butyrate, were protective against microglial dysfunction and reduced trauma-associated anxiety-like behavior, whereas germ-free mice exhibit profound microglial defects.^15^^,^^112^ Bifidobacterium strains commonly found in the normal infant microbiota (B. longum subsp. infantis, B. breve, B. bifidum, B. dentium) restored microglial function and normalized synapse-promoting gene expression.^113^ These bacteria are less abundant in undernourished children.^114‐116^

At the molecular level, aberrant microglia upregulate DDIT4/REDD1, a nutrient-sensing regulator that inhibits mTORC1, a key growth and metabolic hub.^15^ In rodent models of nutrient deprivation, elevated DDIT4/REDD1 suppresses mTORC1 signaling, whereas prolonged refeeding lowers DDIT4/REDD1 and restores mTORC1-dependent protein synthesis.^117^ SCFA supplementation normalizes DDIT4/REDD1 expression in germ-free mice and rescues microglial morphology, demonstrating that microbial metabolites can re-engage nutrient-sensing pathways disrupted by undernutrition, thus identifying a potential therapeutic target.

(b) Oligodendrocytes and Myelination

During development, mature oligodendrocytes myelinate axons and provide metabolic and trophic support. This is a highly energy-demanding process that requires substantial ATP for lipid production and membrane assembly.^118^ Undernutrition or altered microbiota input disrupts this process.^10^^,^^70^^,^^119^^,^^120^ Pathology studies indicate that key myelin components, such as phospholipids and sphingomyelin, are reduced in malnourished children, particularly in the second year of life.^121‐123^ The gut microbiota is a potent regulator of myelin development. Myelination-related genes are upregulated in the prefrontal cortex, and myelin sheath thickness is increased in GF mice, indicating that microbial signals influence myelination timing rather than oligodendrocyte proliferation.^70^ Antibiotic disruption of the microbiota in early life leads to altered oligodendrocyte development, dysregulated myelin gene expression, cognitive defects, and anxiety-like behaviors. These deficits were largely reversed by supplementation with the SCFA butyrate.^124^ Clinical trials show that supplementation with the fatty acid docosahexaenoic acid (DHA) increases plasma DHA and improves gross motor and social-cognitive outcomes in malnourished children.^125^

### Microbiota profiles and cognitive function in healthy subjects

Multiple studies have linked infant gut microbiome profiles to early cognitive outcomes, though findings vary by developmental stage and cohort design. Studies in early infancy report mixed associations. In a pilot study of 40 Canadian infants aged 4–6 months, no significant correlation was found between microbial alpha- or beta-diversity and joint attention performance.^126^ However, successful subtest performance correlated with increased Bifidobacteria, Eggerthella, and SCFA-related metabolic pathways. Interestingly, Bifidobacterium was negatively correlated with EEG rhythm-tracking, suggesting complex roles in neurodevelopment. Similarly, the RESONANCE cohort followed 381 U.S. children from 40 d to 10 y.^127^ No single taxon predicted cognition in the 0–6-month window, but early-life patterns enriched with Enterobacteriaceae and B. longum were associated with later cognitive performance. Further analysis of RESONANCE data identified specific bacterial species associated with brain regions and cognitive abilities.^127^ Across the study period, A. celatus and E. lenta showed strong positive associations with composite cognition. Other species, including B. ovatus and B. fragilis, correlated with cognition-related brain structures, while B. wexlerae and A. hadrus predicted thalamic development and expressive language. F. saccharivorans and S. salivarius emerged as top predictors for gross motor and visual reception scores.

Carlson et al. were the first to show a direct relationship between microbiota composition and cognitive development in late infancy.^128^ In 89 U.S. infants followed from age 1 to 2, those with Bacteroides-dominant microbiomes scored highest on cognitive tests, particularly in language. In contrast, Faecalibacterium dominance and higher alpha-diversity, usually a marker of microbial maturity, were linked to lower cognitive scores. This counterintuitive finding held after controlling for confounders such as birth method and breastfeeding.

In a cross-sectional study of 398 toddlers in rural China, found that alpha diversity did not correlate with Bayley Scales outcomes.^129^ However, higher abundances of major SCFA produces Faecalibacterium, Sutterella, Oscillibacter, and Alistipes, and lower abundances of Clostridium XVIII and Blautia, were associated with better cognitive and motor performance at age 3, accounting for 12% and 24% of mental and motor score variability, respectively.

Complementing these findings, Sordillo et al. analyzed microbiota in 309 U.S. infants aged 3–6 months in a randomized controlled trial.^130^ Gut profiles assessed with the Age and Stage Questionnaire-3 (ASQ-3) predicted neurodevelopmental outcomes at age 3. High Bacteroides levels were linked to poorer fine motor skills, whereas Lachnospiraceae and other Clostridiales taxa (with low Bacteroides) were associated with weaker communication and social interaction.

### Microbiota profiles in undernutrition and selective neurological conditions

Early evidence for a role of the microbiota in childhood undernutrition in mediating neuropsychiatric risk comes from cohorts in Bangladesh and Madagascar.^131^^,^^132^ Portlock et al. analyzed the microbiota of 1-y-old Bangladeshi infants suffering from moderate malnutrition, alongside brain markers such as EEG and the Bayley Scales of Infant and Toddler Development.^131^ Malnourished children had reduced alpha diversity, with elevated oral-associated taxa such as *Rothia mucilaginosa* and *Streptococcus salivarius*, a higher Prevotella/Bacteroides ratio, and more specifically reduced *Bacteroides fragilis,* though not significant. EEG analysis showed that the malnourished group had reduced activity associated with higher cognition, alertness, and concentration, while psychometric data showed reduced communication and motor skills. This finding is consistent with findings from BNS and JS, which found reduced cognition, attention, and motor skills in previously malnourished children.^39,41,44,56‐60,56^ Plasma lipidomics revealed a broad depletion of plasma lipids associated with processes related to glial development and myelination, including lysophospholipids (lysophosphatidylcholine), sphingolipids (ceramides and glycosphingolipids), and alterations in polyunsaturated fatty acids. Specific taxa such as *Bacteroides fragilis* were linked to lipidomic and fermentation pathways involved in communication-related metabolites in healthy children. These findings suggest a potential association between the microbiota and neurodevelopment in ECM via neuroactive lipids produced by the gut.

Conversely, a Malagasy cohort of stunted children over 2 y showed that the main predictors of neurodevelopment were anthropometric measurements of height-for-age Z-score (HAZ) and socio-economic status.^132^ The microbiota alpha diversity was only modestly associated with neurodevelopment scores, while Streptococcaceae was inconsistently associated with personal–social scores. The different findings of this study compared with those of Portlock et al. and many other malnutrition–microbiota studies could plausibly reflect the closure of a sensitive period in microbiota development. Indeed, by age 2, the microbiota approaches a more stable, adult-like state, and most malnutrition studies reporting differences primarily sample children within the first 2 y of life during this sensitive window.

Mounting evidence also links microbiome alterations to autism spectrum disorder (ASD). The Early Autism Sweden (EASE) project revealed that high-risk infants followed distinct trajectories of microbiota development compared with low-risk peers.^133^ In related work, higher alpha diversity was paradoxically associated with poorer cognitive performance in infants suspected of having ASD,^128^ whereas worsening ASD-related behaviors correlated with increased beta diversity, reflecting greater microbial compositional shifts.^134^ The ABIS cohort further reported that infants who developed ASD had a reduced abundance of inflammation-regulating and gut-barrier-supporting bacteria, implicating immune dysregulation in the gut–brain axis.^135^^,^^136^ Microbiome dysbiosis has also been implicated in neurodegenerative and cognitive disorders. A systematic review linked Parkinson's disease to increased abundance of Bifidobacteriaceae, Lactobacillaceae, Rikenellaceae, and Verrucomicrobiaceae, with reductions in beneficial families such as Prevotellaceae, Lachnospiraceae, and Faecalibacterium.^137^ Another review of 10 studies associated taxa such as Haemophilus, Bacteroides, and Alistipes with cognitive decline.^138^

Emotional well-being also correlates with microbiota composition. A systematic review of 25 studies^139^ revealed that Prevotella-dominant profiles were linked to better mood. A greater abundance of SCFA-producing genera (e.g., Agathobaculum, PAC001043g) was associated with lower negative affect, whereas Collinsella correlated with reduced positive affect. Elevated Peptostreptococcaceae was associated with anxiety, whereas Anaerostipes, Porphyromonadaceae, and Parabacteroides were linked to reduced depression and stress. These associations are moderated by sex, dietary fiber intake, and early-life animal exposure.

Lower fecal butyrate and propionate are among the most reproducible metabolomic signatures in malnutrition cohorts; these deficits align with the loss of butyrate-producing taxa.^22,24‐26^ Plasma and fecal metabolomics reveal perturbations in amino acid profiles (e.g., decreased essential amino acids or altered bile acid pools) that mirror impaired host nutrient status and microbiota metabolic capacity.^20^^,^^22^^,^^95^ In several studies, combined taxonomic and metabolomic indices (e.g., microbiota maturation index, SCFA levels) correlate more strongly with anthropometry and clinical recovery than taxonomy alone.^17^^,^^22^^,^^140^

### Towards causality: studies in mouse models

In a landmark study, fecal microbiota from undernourished Malawian infants were transplanted into germ-free mice, resulting in significantly reduced lean body mass gain compared to recipients of microbiota from healthy peers.^20^ The undernourished donor microbiota further induced alterations in bone morphometry, liver and muscle metabolism, and dysregulated gene expression in pathways critical for growth and cognition. Notably, bacterial taxa associated with these phenotypes overlapped with age-discriminatory taxa known to emerge during early human development, underscoring the developmental role of the microbiota in shaping both systemic physiology and neural function.^20^

Transplantation of microbiota from 6- and 18-month-old undernourished Malawian donors into germ-free mice maintained on a Malawian diet resulted in impaired growth. When these mice were cohoused with mice colonized by healthy donor microbiota, growth stunting was prevented. Supplementation of the undernourished donor microbiota with Ruminococcus gnavus and Clostridium symbiosum enhanced growth and metabolic outcomes, including those associated with brain function, in recipient animals. These results indicate that microbiota immaturity represents a potential therapeutic target and support a causal relationship between immature microbiota and growth retardation during early-life malnutrition.^23^

Further support for a causal role of the microbiota in neurodevelopment comes from an Italian birth cohort study. Five-year-old children were stratified by cognitive performance using the Griffiths Mental Development Scales, and their microbiota were transplanted into germ-free mice. Strikingly, the cognitive performance of recipient mice mirrored that of the donor children, providing direct evidence that early microbiota composition can shape neurocognitive outcomes. Metabolomic profiling showed that microbiota from high-functioning children produced neuroprotective metabolites, while dietary analysis indicated higher consumption of legumes, eggs, iron, zinc, and vitamin D in this group, highlighting nutrition as a key upstream modulator of microbiota composition and its developmental effects.^141^

Fecal-oral transmission often occurs in unsanitary conditions. A study replicated this in mice fed a low-fat, low-protein diet isocaloric with controls. This was supplemented with repeated exposure to fecal bacteria (MAL-BG model). Microglia displayed abnormal morphology, altered gene expression, and increased phagocytic features in MAL-BG mice. In addition to growth stunting and gut dysbiosis, MAL-BG mice displayed cognitive deficits, notably impaired learning plasticity in the MWMT. Metabolomic analyzes of brain tissue revealed disrupted polyunsaturated fatty acid (PUFA) metabolism and increased lipoxidative stress, implicating altered lipid pathways. These changes occurred in the absence of inflammation and disruption of the blood‒brain barrier. Dietary supplementation with omega-3 PUFAs and antioxidant vitamins (PAO diet) improved cognitive performance in MAL-BG mice. This study identifies altered fatty acid metabolism as a mechanism contributing to cognitive decline.^142^

Taken together, these human‒mouse transfer models establish a role for the maldeveloped microbiota as a driver of developmental delay in the ECM. These findings also support a putative role for this microbiota in brain dysfunction following ECM. Together, these findings provide a rationale for interventions that target the gut microbiome to prevent or remediate developmental impairments.

### Nutritional and microbiota-directed interventions in early childhood malnutrition

A growing body of research has examined whether nutritional or microbiota-targeted interventions can restore healthy microbial development and ameliorate the consequences of early childhood malnutrition. Overall, findings indicate that caloric repletion alone is often insufficient, whereas interventions that promote durable microbiota maturation show greater promise.^143^

Evidence from early supplementation trials suggests limited remediation once growth faltering is established. In the JS cohort, provision of a milk-based formula (~750 kcal and 20  g protein/d) to undernourished children for 15 months improved developmental scores transiently, but performance plateaued below that of nonstunted peers, with no detectable benefit in IQ or memory at 17–18 y of age.^33^^,^^45^ In contrast, the INCAP Nutrition Trial in Guatemala reported that early-life protein–energy supplementation was associated with improved adult mental health and reduced psychological distress.^144^ However, the absence of verified baseline nutritional status and untreated controls suggests that these benefits reflect prevention rather than reversal of neurodevelopmental impairment.

Outcomes in severe acute malnutrition (SAM) have been less favorable. In Bangladeshi children treated with ready-to-use therapeutic foods, anthropometric recovery occurred, but microbiota immaturity persisted, normalizing only transiently during treatment.^17^ Similarly, in the SHINE trial's Environmental Enteric Dysfunction substudy, conventional nutritional interventions had minimal effects on microbiome composition, although microbial metabolic pathways modestly predicted growth.^145^ The BEED study extended these observations by demonstrating that children with persistent growth failure despite supplementation harbored marked small-intestinal dysbiosis; transplantation of a defined microbial consortium from affected children into gnotobiotic mice induced enteropathy, establishing a causal role for microbiota dysfunction.^146^

More encouraging results have emerged from microbiota-directed complementary foods (MDCFs). In randomized trials of Bangladeshi children with moderate acute malnutrition, MDCFs promoted greater enrichment of age-appropriate taxa – particularly Faecalibacterium prausnitzii and other Clostridiales – than conventional rice–lentil supplements, while suppressing markers of microbiota immaturity.^22^ A longer intervention demonstrated faster nutritional recovery, reduced inflammation, and favorable plasma proteomic signatures linked to bone and brain development, alongside enrichment of multiple growth-associated taxa, including Prevotella copri.^140^

Kau et al. showed that transferring IgA-targeted gut microbes from the fecal matter of undernourished children into germ-free mice triggered the immunometabolic response associated with undernutrition, such as a weight loss phenotype, impaired gut epithelial function, microbial translocation with a rise in pro-inflammatory cytokines such as IL-6 and IL-10 that contribute to neuro-inflammation.^21^

Results from probiotic and synbiotic interventions have been mixed and context-dependent. Community-based administration of Lactobacillus acidophilus–fermented curd in India improved growth and reduced diarrheal burden,^147^ whereas a large randomized trial in Malawian children with SAM found no growth benefit and increased gastrointestinal side effects, possibly reflecting impaired colonization in the setting of routine antibiotic use.^148^ By contrast, trials in Congolese and Bangladeshi infants with uncomplicated malnutrition showed that supplementation with Lacticaseibacillus rhamnosus GG, Limosilactobacillus reuteri, or Bifidobacterium longum subsp. infantis improved growth indices and reduced intestinal inflammation, although durable colonization remained incomplete.^115^^,^^149^

Mechanistic animal studies provide essential context for these clinical findings. Colonization of germ-free or antibiotic-treated mice with microbiota from malnourished children induces growth impairment, metabolic abnormalities, and intestinal pathology, whereas transplantation of microbiota from healthy donors or defined consortia rescues growth.^23^ Diet-induced malnutrition in mice similarly results in loss of fermentative taxa, expansion of mucin-degrading taxa, and reduced short-chain fatty acid production – changes that are partially reversed by microbiota-directed foods.^22^^,^^23^

Current data from intervention studies do not demonstrate complete reversal of the ECM phenotype. This reflects the fact that the microbiota does not operate in isolation. It interfaces dynamically with nutrition, infection, inflammation, psychosocial stress, and socioeconomic context.

## Discussion

ECM has been robustly associated with neurodevelopmental issues in human cohort studies. Reports of therapeutic benefit in reducing stunting in humans, together with some evidence of impact on brain-relevant metabolism imply, but do not prove, a causal role for the microbiota in the development of neuropsychiatric disorders.

Prenatal and early postnatal undernutrition is associated with delayed gut microbiota development, loss of SCFA-producing bacteria, and altered microbial functions in infancy and childhood. These changes occur during key stages of brain, immune, and stress development, and are associated with later cognitive, emotional, and psychosocial issues. Although there are clear temporal and biological links, human evidence is mainly correlational due to confounding factors, ethical constraints, and difficulty in precisely manipulating early microbial exposure.

A central question in the field is whether the relationship among early childhood malnutrition, disruption of the gut microbiota, and subsequent brain dysfunction reflects causal biological pathways or an epiphenomenal association arising from shared environmental adversity. Human observational studies, by necessity, remain largely associative; however, when integrated with increasingly sophisticated experimental data from animal models, a coherent framework is emerging in which the microbiota functions as a developmentally active mediator, rather than a passive correlate, of neurodevelopmental risk. Germ-free, antibiotic-perturbed, and gnotobiotic mice demonstrate that disrupting microbiota-derived immune, metabolic, and neuroendocrine signals early in life causes lasting effects on microglial maturation, synaptic refinement, myelination, BBB integrity, and behavior. Human-to-mouse microbiota transfer studies show that microbiota from malnourished children can transmit growth failure, metabolic issues, intestinal problems, and neurobehavioral traits. Microbial consortia or microbiota-based foods can mitigate these effects.

**Figure 1.**
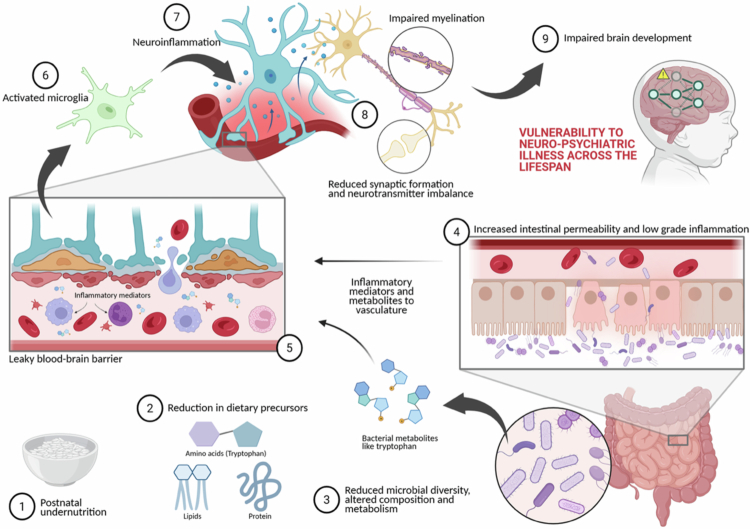
Schematic representation of proposed mechanisms underlying the role of microbiota in altered brain function following ECM. Courtesy of Biorender.

Important caveats limit direct extrapolation to humans. Murine neurodevelopmental timelines compress human prenatal and infant processes into postnatal windows; reduced microbial community complexity may exaggerate effects; and behavioral tests in mice only proxy human cognition and executive functions. Nevertheless, murine studies generate concepts that guide human studies. For example, maldevelopment of the microbiota following ECM may establish a lifelong vulnerability to neuropsychiatric problems that becomes evident later in life in response to environmental or psychosocial cues. In addition, nutritional and microbial changes are often linked, making it hard to attribute effects solely to microbial signaling. Murine studies can provide insight into these issues (Figure 1).

Further investigation is required to determine the role of the microbiota in the susceptibility of malnourished infants to neuropsychiatric disorders and to identify interventions based on improved characterization of the microbiota in cohorts subjected to ECM that later exhibit signs of neuropsychiatric disorder. Establishing a registry and database of these cohorts is encouraged to facilitate cross-referencing of microbiome analyzes and other data. A registry would also facilitate long-term follow-up to identify late-onset neuropsychiatric disorders, as ECM-induced persistent changes in the microbiota,^17^ generate long-term susceptibility to these disorders triggered by environmental and psychosocial events. Future progress will depend on well-characterized birth cohorts with frequent microbiome sampling, rigorously designed early-life interventions, and the identification of biomarkers that link microbial activity to brain development.

Future studies involving malnourished human microbiota-associated mice will provide proof of concept regarding issues such as the onset and duration of brain dysfunction, as well as identifying windows of opportunity for prevention and therapeutic intervention using microbiota-directed therapies. These approaches are essential for determining whether restoring the microbiota can alter neurodevelopmental outcomes rather than solely improving physical growth in children who have experienced ECM.
